# The Role of Pharmacists in Delivering Pharmaceutical Services to Breast Cancer Patients in Clinical and Community Settings: A Scoping Review

**DOI:** 10.3390/pharmacy13040097

**Published:** 2025-07-21

**Authors:** Yuyao Pei, Ruoxin Huang, Feng Chang, Yuanhui Hu, Sarah Versteeg, Yufen Zheng

**Affiliations:** 1 School of Basic Medicine and Clinical Pharmacy, China Pharmaceutical University, Nanjing 210009, China; peiyuyao@cpu.edu.cn (Y.P.); ruoxin23huang@163.com (R.H.); huyuanhui@stu.scu.edu.cn (Y.H.); 2 School of Pharmacy, University of Waterloo, Kitchener, ON N2G 1C5, Canada; feng.chang@uwaterloo.ca (F.C.); sarah.versteeg@uwaterloo.ca (S.V.)

**Keywords:** pharmaceutical care, breast cancer, clinical pharmacist, community pharmacist

## Abstract

(1) Background: Patient-centered care for individuals with breast cancer requires multidisciplinary cooperation to ensure the appropriate use of medication and prevent medication-related problems. Pharmaceutical care has been associated with improved adherence in breast cancer management, a factor linked to patient outcomes and mortality. This study aims to summarize and explore the provision and utilization of pharmaceutical services for breast cancer patients by pharmacists. (2) Methods: A scoping review was performed to assess the pharmacist’s role in providing pharmaceutical services for patients with breast cancer. A comprehensive review of four databases (PubMed, Ovid Embase, Ovid International Pharmaceutical Abstracts, and Scopus) was completed between 1 January 2012 and 8 April 2025 according to PRISMA-ScR framework. (3) Results: A total of 46 articles met the inclusion criteria, which included RCTs, observatory studies, cohort studies, and reviews. Findings suggest that both clinical and community pharmacists play an important role in prevention, management, and education for breast cancer patients. (4) Conclusions: Pharmacists can improve health outcomes by providing pharmaceutical service in breast cancer care. Optimizing interventions, expanding services, and evaluating long-term cost-effectiveness is needed in the future.

## 1. Introduction

Breast cancer (BC) has become the most commonly diagnosed cancer worldwide [1] with an estimated 2.3 million new cases in 2020 [2]. Although treatment varies according to the subtype of breast cancer, some patients receive maintenance pharmaceutical therapy at home for years, similarly to those with chronic disease like diabetes [3,4,5]. For example, adjuvant endocrine therapy for estrogen-positive breast cancer commonly lasts for 5–10 years [5]. This epidemiological landscape, characterized by a high incidence, the need for long-term treatment, and low adherence rates poses a significant challenge [6]. Patient adherence is key to successfully administered long-term treatment. Medication education, which can improve compliance, from a readily accessible source such as a local pharmacists could and has been shown to be beneficial in improving health outcomes [7].

Previous studies have confirmed the necessity and benefit of services provided by pharmacists [8,9]. Such examples include the identification of adverse drug reactions (ADRs) as part of pharmacists’ monitoring plan [10], and their ability to create personalized medication treatment plans for the geriatric high-risk population at different physiological or pathological stages to manage medication side effects and further ensure the best therapeutic outcomes [11]. Mekonnen [9] reported that pharmacist-led medication reconciliation during hospital admission and care transitions significantly reduces ADR-related readmissions and emergency department revisits for all causes. By communicating with doctors regarding therapy adjustment recommendations, pharmacists can improve the prognosis and quality of life (QOL) of patients [12]. Medication reconciliation and suggested adjustments of treatment plans from pharmacists ensure avoidance of harmful drug–drug interaction (DDI) and therapy side effects [13]. To facilitate patients’ active involvement in therapy and enhance medication adherence, pharmacists can counsel patients on symptoms and signs that facilitate hospital visits, prompt patients to take medicine as prescribed, encourage patients to adopt a better lifestyle, and have a basic understanding of disease and medicine [14,15,16]. Pharmacists can assist patients closely with treatment participation, aid in new therapy initiation, and ultimately improve the therapy effectiveness and adherence [17]. Thus, pharmacists are an important part of the multidisciplinary team [18].

Despite the array of research conducted on BC treatment, the battle against malignancy still faces challenges. For example, there is high heterogeneity in response to medication among patients with different BC subtypes, which could be linked to suboptimal matching of treatment medication [19]. Side effects of BC anticancer therapy, as well as the withdrawal of BC treatment, can be severe and can include nausea, vomiting, alopecia, fatigue, and premature menopause, leading to a significant decrease in QOL [20]. Long-lasting comprehensive monitoring and ongoing evaluation of anti-tumor medication from a pharmacist can be essential for effective treatment and QOL. Pharmacists, especially those in a community setting, can also offer comprehensive medical support for BC prevention, like education and training on breast self-examination (BSE) and medication counseling [21]. It is worth noting that community pharmacists do not have the ability and authority to review the prescription of antineoplastic drugs under China’s current policy. However, the development of direct-to-patient (DTP) pharmacy provides a potential space for community pharmacists to aid in the management of antineoplastic drugs. With emerging opportunities such as these, a critical review of the integrative and individual pharmaceutical services that serve and benefit BC patients can be of great benefit in the battle against malignancy. Thus, the objective of this scoping review was to explore pharmacists’ interventions that could improve pharmaceutical care for BC patients across all pharmacy settings (community, clinic, and hospital), exploring the similarities and differences in those settings and if/how cooperation with pharmacists could benefit BC patients.

## 2. Materials and Methods

**Figure 1:**
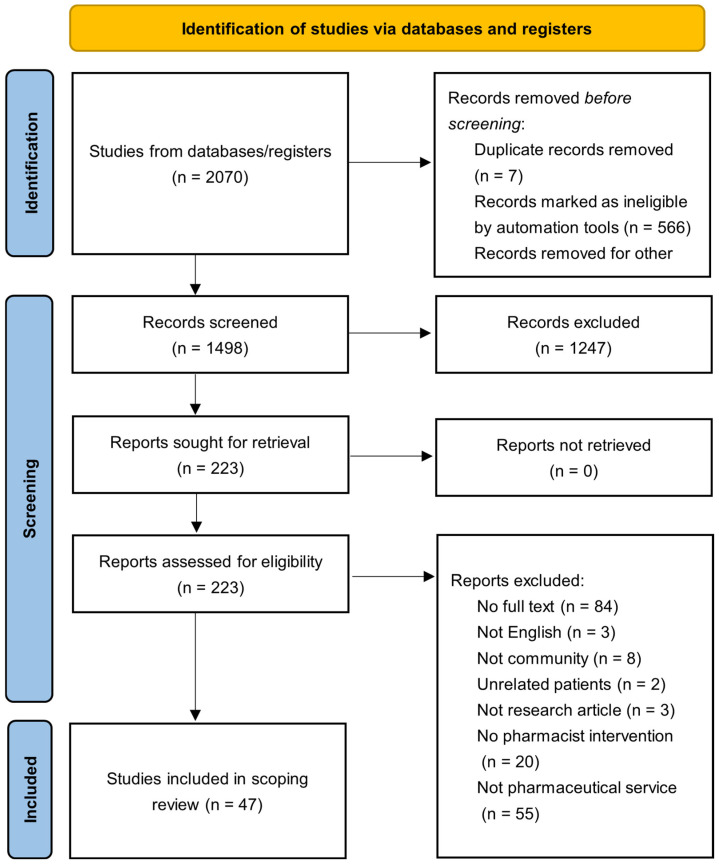
PRISMA flowchart for study selection.

A search strategy was developed to identify studies that included pharmaceutical care for BC patients and the role(s) that pharmacists have in the BC treatment space. To identify pharmacy practice related to BC patients in clinic, hospital, and community pharmacies, the keywords pharmacist/s, pharmacies, pharmacy, pharmaceutical care, pharmaceutical service/s, and breast neoplasms [mesh] were used. The strategy was equally applied to PubMed (MEDLINE), Ovid Embase, Ovid International Pharmaceutical Abstracts, and Scopus from 2012 to 2025 (Appendix A.2). The gray literature was not searched. Search keywords were also applied to Google Scholar, ProQuest Dissertations and Theses Global, and International Pharmaceutical Abstracts (IPA). The study selection process, including identification, screening, eligibility, and inclusion stages, is illustrated in Figure 1 accordance to the PRISMA (Preferred Reporting for Systematic Reviews and Meta-Analyses) guidelines (Appendix A.1) [22]. The protocol was retrospectively registered in the Open Science Framework (OSF) on 11 July 2025 (Available at: http://osf.io/wzqyp/).

Two authors independently scanned the title and abstract to evaluate relevance. The inclusion criteria consist of the following: (1) interventions conducted by a clinical or community pharmacist; (2) articles focusing their research on BC patients or pharmacists engaged in providing services related to BC; (3) all study types, with Chinese and English articles accepted, excluding those without full texts. Recommendations and guidelines were excluded. Reference lists of all included studies were searched for potentially relevant studies.

Retrieved articles were uploaded and managed using Covidence (www.covidence.org, accessed on 25 April 2025), including screening and data extraction. The automatic de-duplication function in Covidence was used and verified by reviewers. Features collected from each article, where applicable, included the country the study was conducted in, study type, pharmaceutical intervention, endpoint evaluation, clinical outcomes, and patients’ subjective and objective experiences with pharmaceutical care.

## 3. Results

### 3.1. Overview

After a review of all databases and reference lists, 2070 articles were identified. After the removal of duplicates, 1499 articles remained. After two rounds of review, 46 articles met the inclusion criteria. In total, 67% of the articles focused on interventions from pharmacists in a hospital or clinical setting (*n* = 31), with nineteen different countries represented across the included articles. Five RCTs were identified, with 82.6% of the articles being observatory or cohort studies (*n* = 38). A total of 87% of the articles discussed detailed pharmaceutical interventions (*n* = 40), and seven studies interviewed BC patients or community pharmacists on attitudes or knowledge. There are five articles (11%) that mentioned virtual pharmaceutical care (e.g., online, phone, etc.). Studies providing only subjective evidence: 13 out of 46 (28.3%). Studies providing objective evidence: 33 out of 46 (71.7%).

### 3.2. Pharmaceutical Service in Hospital or Clinic

Results from the 31 articles that were specific to pharmaceutical services in hospital or clinical settings indicate that pharmacists play a crucial and multi-faceted role in various aspects of BC care. A summary of all these articles is listed in Table 1.

#### 3.2.1. Medication Therapy Management (MTM)

MTM was a focus in four studies. The role of clinical pharmacists in MTM included reviewing medications, comorbidities, referring to multidisciplinary teams, education, and the identification of toxicity. All four studies showed positive change on clinical outcomes [24,30,44,53]. There was only one study that mentioned the billing of MTM, in which a ‘no charging standard’ was established [44].

#### 3.2.2. Patient Education and Adherence Improvement

Nearly half of these hospital- or clinic-based studies (*n* = 14) discussed the application and benefit of patient education. Verbal expression [39], written documents [40,52], and online platforms [29] were effectively used to offer knowledge of BC and its affiliated treatments. The education provided by pharmacists encompasses the following aspects: medication tables [40], prevention and management of ADRs [29,42], information on BC, the necessity of therapy [32,34,46], interventional strategies for anticipated adverse events [36,42], medication guide [29,51], handling of missed doses [34], lifestyle recommendations (dietary advice) [51,52], and pharmaceutical counseling [27,50].

#### 3.2.3. Optimization of Drug Therapy

Medication reconciliation [23,25,45,50], multidisciplinary collaboration and care coordination [26,31,38,45], deprescribing, and prevention of DDI [32,35,43,47] were commonly found among these hospital- and clinic-based articles. These articles showcased how pharmacists in these settings were able to identify potential and current DRPs, document changes in medicine, suggest alternative treatment plans when necessary, and keep the doctor informed in a timely manner [31]. The result of laboratory examinations was also part of a shared pharmacy care model [28]. The management of symptoms and ADRs was shown to enhance the adherence of BC patients and improve the safety of the treatment used [27].

#### 3.2.4. Quality of Life and Humanistic Outcomes

These articles also showed improvement in QOL [32,46], patients’ satisfaction [37], and humanistic outcomes such as patient mobility and physical function, activities of daily living, instrumental activities of daily living, pain and discomfort management, and negative emotions [38].

### 3.3. Pharmaceutical Service in Community

Fifteen articles that were specific to pharmaceutical services in a community setting were identified. A summary of all these articles is listed in Table 2.

#### 3.3.1. Health Education and Prevention

In total, 60% of these identified articles engaged community pharmacists in the delivery of education to BC patients (*n* = 9). Examples included disease education [57], BSE [59], breast examination and assessment [55], health education booklets [65], and enhancing patients’ understanding of the rehabilitation process [68]. Prevention strategies shared by pharmacists included education on BC symptoms, risk factors, methods of detection and diagnosis, prognosis, and severity of breast cancer [59,65].

#### 3.3.2. Medication-Related Services

These articles also showcased community pharmacists offering online platforms for comprehensive medication-related services [57], cooperation with medical teams to optimize therapy plans [54], consistent follow-up [56], management of AEs [60,63], and extended services and community care [60,62,67,69]. These articles also showed that pharmacists in community settings intervene in cases of AEs, DDIs, and side effects, embrace the identification of potential adverse reactions [54], manage side effects [63], and report AEs. Other forms of care provided by community pharmacists in these articles included counseling around weight management and lifestyle modification [62], the evaluation of dosage adjustment [67], and implementing medication delivery during the COVID-19 pandemic [60].

#### 3.3.3. Attitude Towards Pharmaceutical Services in Community

Two articles captured the attitude towards pharmaceutical services in communities [58,61]. The lack of knowledge about cancer, the focus on healthcare issues other than cancer, and the perceived role of pharmacy staff were identified as the greatest barriers preventing staff from providing comprehensive consultations to cancer patients/survivors [58]. It was suggested that incentives are used to showcase the value of pharmacists’ roles in caring for BC patients [61].

## 4. Discussion

This scoping review synthesized evidence from 31 studies on hospital/clinic pharmacists and 15 on community pharmacists, comprehensively elucidating their roles in delivering pharmaceutical services to BC patients. The findings highlight the far-reaching benefits of pharmacist-driven and pharmacist-engaged interventions and underscore the crucial significance of MTM in breast cancer care.

### 4.1. Benefits of Pharmacist Services for Breast Cancer Patients

Pharmacists are integral in enhancing medication adherence among BC patients through strategies such as personalized counseling, reminder systems, and educational materials. These strategies can enhance adherence, directly impacting treatment effectiveness and patient prognosis. For example, the included clinical pharmacist-led patient education and follow-up intervention conducted in Iraq notably optimized breast cancer patients’ adherence to adjuvant hormonal therapy [40]. This outcome was also found in an included Canadian study, where pharmacist-provided pharmaceutical care, including medication counseling and follow-up, increased patients’ adherence to CDK4/6 inhibitors [56]. Supporting adherence can help avoid treatment failure and disease recurrence, highlighting the importance of this outcome.

Pharmacists contribute significantly to augmenting patients’ knowledge about breast cancer and its treatment. For example, in an included study conducted in Egypt, it was demonstrated that community pharmacists’ teaching significantly increased knowledge of breast cancer prevention and preventive actions [59]. Ramzi et al. found that community pharmacists in a Palestinian context played a role in warning people about breast cancer risks and prevention methods [64]. Well-informed patients are more likely to actively engage in their treatment, make well-informed decisions, and adhere to treatment plans.

Pharmacists play a key role in managing the side effects of breast cancer treatments. Colombo et al.’s systematic review in Brazil indicated that pharmacist interventions, mainly centered on educating and counseling patients on adverse event management, could improve outcomes in cancer outpatients [54]. In the Netherlands, Selma et al. found that women on adjuvant endocrine therapy desired more side effect management education from pharmacists [63]. By providing information on side effect prevention and management, pharmacists can enhance patients’ quality of life during treatment.

Hospital and clinic pharmacists are essential in optimizing drug therapy. A.C. Ferracini’s cross-sectional, prospective study in Brazil showed that clinical pharmacist reviews of patients’ medical records and prescriptions, along with interventions when prescribing errors were detected, avoided significant prescribing errors [25]. In Spain, Carmen’s prospective study demonstrated that hospital pharmacists’ systematic review of treatment, detection of interactions, and recommendations reduced medication-related problems and optimized drug therapy [47]. This ensures that patients receive safe, effective, and appropriate medications.

Pharmacist-led interventions have a positive impact on patients’ quality of life. Avinash et al.’s study in India showed that the provision of oncology pharmacist services led to an improvement in QALY [38]. In Japan, Kazuhide’s cohort study found that personal counseling by pharmacists improved the treatment environment and enhanced the quality of life of breast cancer patients [42]. By addressing various aspects of treatment, such as medication-related issues, side effect management, and patient education, pharmacists contribute to a better overall quality of life for patients.

### 4.2. Significance of Medication Therapy Management (MTM) Services in Breast Cancer Care

In the realm of breast cancer care, MTM services hold significant importance. These services foster multidisciplinary collaboration, as evidenced by Pedro’s study in Brazil, which showed that oncology-focused MTM involved referrals to multidisciplinary team members. In the United States, Koshy’s research on the Cancer and Aging Interdisciplinary Team (CAIT) clinic model further demonstrated the crucial role of collaboration between pharmacists and other healthcare providers [49]. As part of the multidisciplinary team, pharmacists bring their specialized medication therapy knowledge, which complements the skills of oncologists, nurses, and other professionals, resulting in more comprehensive patient care. MTM services are also essential for identifying and resolving DRPs. Jianping Zhang’s retrospective study in China on an independent anti-neoplastic MTM system revealed that it effectively facilitated the identification and resolution of DRPs, with a particular emphasis on improving medication adherence [30]. Minoh’s retrospective analysis in a Korean hospital found that the collaborative deprescribing service within the Consultation-Based Palliative Care Team (CB-PCT) was successful in identifying and addressing medication-related issues. By detecting and resolving DRPs, pharmacists can prevent adverse drug events, optimize drug therapy, and enhance patient safety [45]. Moreover, MTM services enable tailored, patient-centered care. Huijie’s study in China on integrated pharmacy services indicated that professional pharmacists, as part of a multidisciplinary team, enhanced compliance and improved cancer treatment outcomes through personalized care [29]. In Switzerland, Carole Bandiera’s RCT on community pharmacists’ medicine management for breast cancer patients, which involved monthly motivational interviews to monitor adherence to palbociclib, serves as an excellent example of personalized care [66]. Tailored care considers patients’ individual needs, preferences, and circumstances during treatment, leading to better treatment outcomes and higher patient satisfaction. Finally, MTM services provided by pharmacists can help alleviate the shortage of oncology providers. Elaine’s observational study in a Canadian hospital showed that integrating pharmacists into a shared-care model reduced the number of ambulatory patient visits for oncologists [28]. By leveraging pharmacists’ medication expertise, healthcare systems can better manage the growing number of breast cancer patients, ensuring that patients receive timely and appropriate care.

### 4.3. Comparison Between Hospital and Community Pharmacists in Breast Cancer

Pharmacists who operate primarily within hospital or clinic settings (also known as hospital pharmacists) have their services directly integrated into the inpatient and outpatient treatment processes. In the studies of Pedro in Brazil and Avinash in India, they worked closely with on-site oncology teams, having immediate access to patients’ medical records, test results, and treatment plans [24,38]. This setup allows in-depth, real-time patient care but is restricted to the hospital’s patient population. Community pharmacists, conversely, offer services in community pharmacies, which are more accessible to the public. Xie Chengsheng’s study in China used a WeChat-based platform to extend services to cancer patients outside the hospital, reaching those who may not frequently visit a hospital [57]. Community pharmacists can interact with patients in their daily lives, facilitating long-term follow-up and continuous care. Hospital pharmacists concentrate on aspects like MTM, the prevention and resolution of DRPs, and ensuring medication safety and efficacy. In a study by A.C. Ferracini in Brazil, they reviewed patients’ medical records and prescriptions to detect and correct prescribing errors [25]. They also play a significant role in multidisciplinary teams. Based on our findings, community pharmacists focus more on health education and preventive care when it comes to BC (Table 3). In Egypt, Osama et al. educated females about breast cancer prevention [59]. They also assist with medication adherence and provide basic side-effect management counseling, though to a lesser extent than hospital pharmacists. For example, Selma et al. in the Netherlands found that women desired more side effect management education from community pharmacists [63]. Hospital pharmacists customize their services based on the complex medical conditions of in-patient and out-patient breast cancer patients. Kazuhide’s cohort study in Japan provided personalized counseling on expected adverse events and correct medication use for supportive therapy [42]. They often handle acute and severe cases, coordinating with other medical staff to optimize treatment plans. Current studies show cooperation between community pharmacists and hospital pharmacists, which reveals a new approach, to ensure BC patient receive a full-process service [67,69]. 

### 4.4. Challenges of Community Pharmacists’ Medication Therapy Management for Breast Cancer Patients

Community pharmacists may face challenges due to limited resources, such as restricted access to comprehensive patient medical records and advanced monitoring tools. Compared to hospital pharmacists, they may also have less in-depth training in oncology-specific MTM. Nazri et al.’s review in Malaysia indicated that although community pharmacists have potential extended services, the lack of specialized training might limit their ability to provide high-quality oncology care. BC patients have diverse needs, and some may have complex medical conditions and comorbidities. Community pharmacists may find it difficult to handle complex cases without the immediate support of a multidisciplinary team, as available in hospitals. For example, Selma et al. in the Netherlands found that women on adjuvant endocrine therapy have specific side effect management needs that community pharmacists may struggle to fully address. Integrating community pharmacy services into the broader healthcare system can be challenging, because economic evaluation (cost–benefit ratio) is awaiting further research [37,69]. There may be limited communication and collaboration channels between community pharmacists and hospital-based healthcare providers. Community pharmacist used email to send reports on evaluations to hospitals, which may not be timely [67]. Strengthening timely information sharing between community pharmacists and medical service providers in hospitals or clinics may be the potential direction for pharmacists to better participate in BC management in the future. This can result in gaps in patient care, such as inconsistent treatment information and a lack of coordinated follow-up. Ensuring the seamless transfer of patient information between hospitals and community pharmacies remains a hurdle to providing comprehensive care for breast cancer patients.

### 4.5. Limitations

Although we searched multiple databases (e.g., PubMed, Scopus, Ovid Embase), we only examined articles written in English and Chinese, which may have led to language bias and the exclusion of relevant findings from other regions. Gray literature papers and preprints were excluded. This study included qualitative, quantitative, and mixed methods studies, which broadened perspectives, but methodological heterogeneity hindered the unified integration of topics. This methodology was appropriate for our objective of mapping existing evidence; however, scoping reviews inherently emphasize breadth over depth, which limits detailed evaluation of intervention effectiveness or comparative outcomes.

## 5. Conclusions

In conclusion, pharmacists play a pivotal role in providing pharmaceutical services to breast cancer patients. Their services offer numerous benefits for therapy and health outcomes, and MTM services are of great significance. However, to further optimize these services, future research should focus on improving pharmacist-led interventions, expanding their reach, and evaluating their long-term cost- effectiveness in BC care. In addition, the quantity and quality of services provided by community pharmacists are far less than those provided by clinical pharmacists, and many community pharmacists are not too active in providing professional services for BC. Therefore, future research on the BC pharmaceutical service management system, while also considering economic factors such as cost-effectiveness and pharmacist revenue to guide the further development of community pharmacists and evaluate their healthcare and economic benefits, may help community pharmacists and clinical pharmacists complement each other and provide the most comprehensive services for patients.

## Figures and Tables

**Table 1:** Summary of studies focused on hospital or clinic pharmacists (*n* = 31).

Last Author Country	Study Design	Clinical Setting	Intervention	Key Findings
Darcis et al., 2023 [23] Belgium	Case–control study	Hospital	The pharmacist performed a medication reconciliation and medication review.	The medication list in the EPR was incomplete in 74% of patients with an average of 2.4 errors per patient. After medication review, the adapted Medication Appropriateness Index score decreased significantly (*p* < 0.001). Acceptance rates were 41% and 53% for medication reconciliation and medication review advice.
Amaral et al., 2018 [24] Brazil	Observational, descriptive, and retrospective study	Oncology ambulatory clinic of a tertiary hospital	MTM services offered to patients with BC in the use of polypharmacy.	A total of 185 DRPs were identified, 48% were resolved, and 50% were in the resolution process.
Ferracini et al., 2018 [25] Brazil	Cross-sectional, prospective study	University Medical Center	A clinical pharmacist reviewed patients’ medical records, monitored patients’ exams, and analyzed aspects of electronic prescriptions. When prescribing errors were found, a pharmaceutical intervention was conducted.	In total, 11.5% of prescriptions analyzed included at least 1 prescribing error. The clinical significance of prescribing errors and interventions were classified as significant and very significant, respectively. The pharmacist performed 294 pharmaceutical interventions, of which 74% were accepted.
Ribeiro et al., 2018 [26] Brazil	Semi-structured interviews	Hospital	CMM service offered by pharmacists.	Six major themes emerged that reflected the process of implementation of CMM services from the pharmacists’ perspective: resistance is human, insecurity with being a clinician, management of change supported by driving forces, pharmaceutical care fosters professionals’ self-efficacy, documentation is the conducting wire of the practice, and the advantages of a systematized practice.
Staynova et al., 2024 [27] Bulgaria	Scoping review	N/A	Impact of pharmacist-led interventions on BC management and health outcomes.	Fourteen studies were included. Pharmacists commonly provided the following interventions: consultations regarding chemotherapy treatment, risk assessment and patient education, adverse drug reactions and drug–drug interaction detection, and adherence assessment. Beneficial effects of the involvement of pharmacists in BC management included better quality of life, reduced drug interaction risk, greater adherence rates, and improved patient knowledge.
Goh et al., 2023 [28] Canada	Observatory study	Hospital	Medication assessment by pharmacist program (oncology pharmacists conducting medication assessment visits in a shared care model).	A total of 46 medication assessments by visiting pharmacists resulted in 920 min of clinic time savings for physicians. In all, 100% of surveyed oncologists felt this intervention reduced workload and wanted the intervention to be expanded to additional oncology drugs. Further, 100% of surveyed pharmacists felt that the intervention increased job satisfaction and allowed further application of clinical skill.
Qi et al., 2021 [29] China	Retrospective study	Hospital	Integrated clinical oncology pharmacists into a new oncology pharmaceutical care program (an online medication management system).	The percentage of patients missing administration every day was reduced from 30% to 0%. Fewer patients in the integrated pharmacy service group visited the clinic and ER compared to the routine care group (33% versus 59%, *p* < 0.05).
Zhang et al., 2021 [30] China	Retrospective study	Hospital	An independent anti-neoplastic MTM with six modules: medication therapy review, intervention plan, personal medication record, medication-related action plan, intervention and/or referral, and documentation and follow-up.	Identified and solved DRPs (85.5%) and improved medication adherence of patients (84–100%).
Novosadova et al., 2023 [31] The Czech Republic	Prospective open-label clinical study	Hospital	Inclusion of clinical pharmacist in the palliative care team, evaluating their contribution towards diminishing or ameliorating the risk of polypharmacy and non-compliance in palliative oncology care patients.	Significant association between drug-related problems and polypharmacy (*p* < 0.001). According to patients’ feedback, the presence of a clinical pharmacist improved the perception of their quality of life.
Farrag et al., 2020 [32] Egypt	Single-center prospective study	Clinical Oncology Department	Patient counseling + educational program (disease specific information) + specific messages related to health motivation, susceptibility to breast cancer, the perceived benefits and barriers of mammography, and perceived self-efficacy.	Significant decrease in toxicity grades of patients and improvement in QOL scales (e.g., decreased systematic therapy side effects, *p* < 0.0001).
ElBaghdady et al., 2024 [33] Egypt	Cross-sectional study	Hospital	No intervention (assessed the adherence to oral hormonal therapy).	A total of 27% of surveyed patients preferred to obtain medication information from pharmacists. When seeking information, 27% of surveyed patients consulted pharmacists.
Feral et al., 2022 [34] France	Retrospective single-center study	Hospital	Multidisciplinary consultation program (oncologist, pharmacist, and nurse).	The intervention group had fewer adverse events, in general, and digestive adverse events in particular as compared to the control group (*p* = 0.048 and *p* = 0.007, respectively).
Leenhardt et al., 2021 [35] France	Prospective clinical trial	Hospital	Hospital pharmacist interview to identify co-treatments and DDI risk. If necessary, treatment changes were made by the pharmacist and oncologist to limit the risk of DDI occurrence.	Pharmacist interventions indicated that at inclusion into the study, current medications were incomplete for 63% of the enrolled patients. The intervention allowed the real-time management of high-risk DDI detected in 1/3 of patients.
Dürr et al., 2021 [36] Germany	Multicenter RCT	Hospital and clinic	Standard care + patient counseling sessions with clinical pharmacologists.	Positive improvements in the number of medication errors, patient treatment perception, and severe side effects (*p* < 0.001).
Liekweg et al., 2012 [37] Germany	Prospective, multi-center cohort study	Community-based outpatient clinics	Application of an algorithm for evidence-based antiemetic prophylaxis, treatment, and medication counseling of patients before and during their courses of chemotherapy. In addition, the patients were counseled regarding the optimal use of supportive medication.	Improved patient-reported outcomes such as emetic episodes, quality of life, and patient satisfaction (*p* < 0.05).
Khadela et al., 2022 [38] India	Prospective, single-center study	Hospital	Provision of anti-neoplastic and supportive care, drug-related information to oncologists, medication chart review, counseling, and educational sessions for para-medical staff by pharmacist.	Improvement in QALYs (pre 0.014 and post 0.043).
Puspitasari et al., 2022 [39] Indonesia	Systematic review	N/A	Impact of pharmacist interventions on medication adherence in patients with cancer taking capecitabine.	Five studies were included. The most common pharmacist intervention strategy was a combination of patient education with oral and written information provided. Pharmacist interventions provided beneficial impacts on medication adherence, beliefs about medication, and tolerability of seif effects.
Rabeea et al., 2023 [40] Iraq	RCT	Hospital	Educational session delivered by a clinical pharmacist + educational sheet + phone-based medication reminder.	There was a 65% adherence rate to oral hormonal therapy, with significantly improved necessity beliefs and necessity–concern differentials when compared to the control group.
Suzuki et al., 2019 [41] Japan	Retrospective cohort study	Outpatient clinic	Clinical pharmacists collaborated with an oncologist in the treatment of adverse drug reactions in outpatient cancer chemotherapy before, during, and after outpatient examination.	Pharmacists provided interventions to 498 cases, with a 79% acceptance rate by the oncologist. In total, 57% of the adverse drug reactions were improved following the pharmacists’ suggested prescriptions.
Tanaka et al., 2018 [42] Japan	Cohort study	Hospital	Pharmacist counseling.	Malaise and nausea were significantly higher in the non-intervention group compared to the intervention group (*p* = 0.043 and *p* = 0.017).
Todo et al., 2018 [43] Japan	Cohort study	Outpatient cancer chemotherapy clinic	Comprehensive pharmaceutical care for prevention of severe AEs.	Maintained QOL heath states after 6 months of intervention (pre 0.850 and post 0.889). Median time to treatment failure was significantly longer after intervention than before (224 days versus 34 days, *p* < 0.001).
Watkins et al., 2012 [44] Japan	Retrospective review	Ambulatory clinic	MTM to augment services already provided.	In a 3-month period, 239 MTM visits were completed, with a 20 min median of face-to-face time and 18 min median for documentation per visit. No claims for MTM were rejected, and reimbursement rates ranged from 47–79%.
Ko et al., 2023 [45] Korea	Retrospective analysis	University Hospital	Pharmacist-led deprescribing service within a consultation-based palliative care team setting was implemented for terminal care patients. This service included medication reconciliation, comprehensive medication evaluation and deprescribing services, delivery of medical information to healthcare professionals, and continuity during discharging of pharmaceutical care services.	Higher deprescribing rates and acceptance rates in the intervention group as compared to usual care (30% vs. 10% *p* < 0.001 and 78% vs. 30% *p* = 0.003, respectively). More intervention patients had one or more MRPs deprescribed as compared to usual care (40% vs. 3%, *p* < 0.001). Clinical significance of the deprescribing service was evaluated as ‘very significant’, with a mean score of 3 out of 4.
Subramaniam et al., 2025 [46] Malaysia	RCT with a single-blind study design	Cancer Institute	‘Improving Quality of Life in BC Patients Undergoing Chemotherapy’ educational module was implemented through repetitive pharmacist counseling as compared to counselling sessions using the hospital’s existing counselling practices.	The intervention was effective in improving QOL and depression among participants at baseline and for 3 consecutive follow-ups and showed significant improvement in all 4 QOL domains (*p* ≤ 0.001). There was also a moderate effect reduction on depression (*p* < 0.001).
Lopez-Martin et al., 2014 [47] Spain	Prospective study	Tertiary hospital	Hospital pharmacists’ recommendations.	The pharmacy service intervened in 83% of cases when patients used alternative medicine. The pharmacist’s recommendations were accepted in 94% of cases.
Birand et al., 2019 [48] Turkey	Interventional prospective study	Tertiary hospital	Counseling by an oncology pharmacist.	Pharmacist education significantly enhanced the mean patient necessity–concern balance scores by two-found (*p* < 0.0001), with patients who received counseling for the first time experiencing the greatest benefit.
Alexander et al., 2023 [49] USA	Prospective clinical trial	Hospital	Utilized a team clinic model (oncologist, geriatrician, registered nurse, pharmacist, and registered dietitian) to provide timely pretreatment geriatric assessment and treatment recommendations independent of patient’s physical location. Role of pharmacist on team: discontinuation, changes or additions of medication, identification of significant interactions, and red flags for non-adherence.	The median days between receiving a referral and having an appointment was 8. GA detected multiple unidentified impairments. A total of 93% agreed that their goals for referring the patients to the team were met and that the clinic helped define an optimal treatment plan. A total of 100% responded that they would refer patients to the team again.
Ganihong et al., 2024 [50] USA	Single-center retrospective chart review	Cancer Center	Clinical pharmacist in supportive care management (chart review of documented pharmacist interventions with patients presenting to the clinic for new chemotherapy).	The pharmacist directly managed 33% of patient-reported adverse drug effects. The pharmacist made 1,068 interventions spanning 190 documented hours across a 6-month time period. Common interventions were coordination of care, education, and supportive care pharmacotherapy interventions.
Homan et al., 2021 [51] USA	Observational study	Hematology/oncology clinic	Provide patient symptom management through CDTM.	A total of 196 categorized pharmacist interventions were captured. Most patients (69%) had a reduction in the severity of their referral diagnosis symptoms.
Patel et al., 2023 [52] USA	Quality improvement study	Medical Center	High-touch pharmacy intervention incorporating pharmacists within outpatient oncology clinic visits with providers. Pharmacists met with patients, identified barriers to treatment, and performed counseling.	Decreased average treatment day delay for patients (*p* < 0.0001). In total, 640 pharmacy interventions were documented, including medication reconciliation and clinical recommendations.
Solomon et al., 2019 [53] USA	Single-center, retrospective chart review	Institutional setting	Education of patients, assisting oncologists and nurses with treatment and therapy plans, ensuring appropriate supportive care options for each patient, and addressing any drug-related questions.	Overall, 27% of patients received education by a clinical pharmacist before initiating therapy.

**Table 2:** Summary of studies focused on community pharmacists (*n* = 15).

First Author, Country	Study Design	Setting	Intervention	Key Findings
Colombo et al., 2017 [54] Brazil	Systematic review	N/A	Examined the effects of pharmacist interventions on adult outpatients with cancer using antineoplastic drugs.	Eleven studies were included. Educating and counseling patients on the management of adverse events were the most common pharmacist interventions included. In most studies, a significant benefit to the rates of nausea and vomiting control, medication adherence, and patient satisfaction were found.
Havlicek et al., 2016 [55] Canada	Review	Community pharmacy	The aim of this review was to examine the pharmacist’s involvement in malignancy screening and prevention, to education pharmacists on the current recommendations for cancer screening, and to explore other potential opportunities for implementing screening services into their practices.	The review suggested that pharmacists could be more involved in malignancy screening and prevention, summarized current screening recommendations and suggested resources, listed risk factors and malignancy prevention strategies, and outlined opportunities for pharmacists. These opportunities included promoting general public awareness on prevention and screening, education on cancer prevention for vulnerable populations, risk assessment and referral for further testing, and risk assessment and testing within the pharmacy setting.
Marineau et al., 2023 [56] Canada	Retrospective chart review	Community pharmacy	Patient-centered pharmacy practice that aimed to improve patient outcomes by minimizing administrative delays in patient treatment, promoting treatment adherence, preventing and managing adverse events, and empowering patients to become a partner in their own care.	Mean of 7 clinical and administrative activities for each 28-day CDK4/6i treatment cycle. Most common activities performed (70%) included direct patient communication and verification of laboratory test results. A mean time-to-treatment initiation of 18.5 days was observed in patients treated with CDK4/6i.
Xie et al., 2023 [57] China	Design a platform to offer management for breast cancer patients	Virtual platform	A multimedia, interactive and whole-process pharmaceutical service system integrating drug information inquiry, information release and pharmaceutical care.	Successfully established the system to include pharmaceutical service functions, including multimedia drug propaganda and education, drug knowledge databases, and patient whole-process management.
Buhl et al., 2023 [58] Denmark	A cross-sectional questionnaire	Community pharmacy	No intervention (study aimed to determine community pharmacy staff’s knowledge, educational needs, and barriers when communicating with cancer patients/survivors).	The most well-known topics concerned risk factors for cancer and side effects from cancer treatment. A lack of knowledge about cancer, a focus on healthcare problems other than cancer, and a traditional view of community pharmacies as a place to pick up medication were the largest barriers identified in counseling cancer patients/survivors. The surveyed pharmacy staff expressed interest in participating in educational programs (91%), communication with cancer patients (88%), and the late effects of cancer (93%).
Ibrahim et al., 2021 [59] Egypt	RCT	Community pharmacy	Pharmacist-based coaching (12 weekly face-to-face coaching sessions)	At the end of the study, there were significantly more patients performing high physical activity, practicing healthy diets, and practicing breast self-exams in the intervention group as compared to the control group (*p* ≤ 0.05). Mean scores of knowledge on BC symptoms, risk factors, and detection methods at 3-month follow-up were higher in the intervention group as compared to the control group (*p* ≤ 0.05). In total, 35% of participants were ‘uncomfortable towards the competency of coaches’.
Larbre et al., 2023 [60] France	A prospective monocentric study	Over the phone	During the COVID-19 pandemic, telephone follow-up was conducted via an Oncoral hospital/community multidisciplinary program where continuity care was maintained by a pharmacist/nurse pair.	During lockdown, follow-up enabled 61 interventions by the pharmacist and nurse for 42 patients. The community pharmacist was involved in 20% of cases, mainly to coordinate drug ordering with the patient or family member coming to the pharmacy (83%). A total of 83% of patients were satisfied by the telephone follow-up established, with 69% in favor of repeating these follow-ups in the case of a new epidemic wave. A toal of 71% felt well involved in the exchanges with the hospital telephone follow-up team.
Gharaibeh et al., 2024 [61] Jordan	A cross-sectional questionnaire	Community pharmacy	No intervention (study aimed to assess the knowledge, attitudes, and barriers of community pharmacists in promoting early detection services).	In total, 38% of female pharmacists over 40 years old underwent a mammogram. Knowledge of symptoms of breast cancer was the highest, followed by knowledge of risk factors and early detection of breast cancer. A lack of educational materials and time constraints were examples of voiced barriers. Higher knowledge scores were associated with factors, including but not limited to geography (*p* = 0.003), gender (*p* < 0.001), and frequency of inquiries by customers (*p* < 0.001).
Nordin et al., 2017 [62] Malaysian	Systematic review	N/A	The aim of this review was to identify actual or potential extended services performed in community pharmacy settings, perceptions among community pharmacists, general practitioners, consumers, and policymakers of these extended services, and barriers towards its performance.	Eight studies were included. Nineteen actual or potential extended services were identified, with medication counseling and conducting smoking cessation programs being the ‘most rated’ extended services. Customers were in favor of community pharmacists performing these identified services; community pharmacists’ indicated barriers to performance of these services and general practitioners’ perceptions were mixed.
En-Nasery-de Heer et al., 2022 [63] Netherlands	A qualitative explorative study	Semi-structured face-to-face interviews	No intervention (assessed the needs and wishes of women using AET regarding pharmaceutical care and eHealth).	Three themes were identified: experience with AET use, experience with provided information, and needs and wishes regarding pharmaceutical care. It was reported that most interviewed women felt pharmacists were ‘hardly involved in providing information on the use of AET’ with some participants indicating that pharmacists could ‘play a more elaborate role, especially at the start of AET’. The voiced ideas on the role of the pharmacist included ‘counseling on a regular basis and informing AET users more comprehensively in a patient-tailored manner’, with a few interviewed women suggesting ‘a more intensive collaboration between pharmacists and other healthcare professionals, such as their general practitioner’.
Shawahna et al., 2021 [64] Palestine	Cross-sectional questionnaire	Community pharmacists	No intervention (assessed knowledge, attitude, beliefs, and barriers towards BC health promotion among community pharmacists).	The median knowledge score was 69%. Overall, 68% of community pharmacists scored ≥ 50% in the knowledge test, with a significant moderate positive correlation between knowledge and attitude scores (*p* < 0.001). In total, >60% of surveyed pharmacist agreed that a lack of reimbursement, lack of enough personnel, lack of time, and fear of offending the patient were barriers to BC health promotion.
Brzykcy et al., 2024 [65] Poland	Review	Community pharmacy	The aim of the article was to identify the role of pharmacists working in community pharmacies in BC prevention, as well as effective health promotion methods that can be implemented in daily practice, resulting in benefits for the healthcare of patients.	Included examples of pharmacists’ roles in BC prevention: educating and following up on breast self-examinations, using leaflets and posters among community pharmacists to build awareness, developing new health promotion programs, selling screening kits to individuals who do not qualify for free screening, and promoting mammography.
Bandiera et al., 2023 [66] Switzerland	RCT	Community pharmacy	A 12-month interprofessional medication adherence program + monthly motivational interviews by a pharmacist.	Face-to-face motivational interviews lasted 18 min on average vs. the control group who met the pharmacist at the pharmacy counter for 8 min on average. Intervention group maintained higher and more stable palbociclib implementation compared to control patients. The intervention did not impact persistence to palbociclib.
Takada et al., 2024 [67] Japan	Single-center, retrospective, observational study	Collaboration between hospital and community pharmacies	Collaborative medication management (collaborative work follow-up between pharmacists and physicians regarding hand–foot syndrome control for capecitabine).	Significantly lower cumulative incidence of ≥Grade 2 hand–foot syndrome was found in the intervention group as compared to the control group (6% vs. 68%, *p* < 0.0001). The pharmacist intervention group presented a significantly prolonged time for the onset of ≥Grade 2 hand–foot syndrome as compared to the control (*p* = 0.006).
Lindsey et al., 2015 [68] UK	Systematic review	N/A	The aim of this review was to identify and assess the current evidence for the role of community pharmacies in delivering early cancer detection initiatives.	Twelve studies were included. Screening was the most commonly identified intervention (83% of studies), followed by education (33% of studies). All studies reported outcome measures related to the domain of behavioral determinants (e.g., awareness, knowledge, etc.); however, heterogeneity in the studies prevented a meta-analysis from being conducted.

**Table 3:** Comparison between the community pharmacist and the clinical pharmacist.

Comparison Aspect	Community Pharmacist	Clinical Pharmacist
Primary focus area	Breast cancer prevention education and promoting preventive actions	Optimizing drug therapy, managing treatment side effects, and addressing prescribing errors
Key responsibilities	Educating females with breast cancer prevention methods	Conducting systematic reviews of patient medical records and prescriptions
	Warning people about breast cancer risks	Detecting medication interactions and providing intervention recommendations
	Assisting with medication adherence	Playing a role in multidisciplinary teams and handling acute and severe cases
Intervention strategies	Providing educational materials on cancer prevention	Offering patient education and counseling on adverse event management
	Promoting preventive actions among the community	Implementing follow-up systems for medication adherence
Impact on patient	Enhancing community awareness and proactive preventive behaviors	Ensuring safe, effective, and appropriate medication use
	Increasing cancer screening participation rates	Reducing medication error risks
		Improving QOL/humanistic outcomes and treatment efficacy
		Improving adherence and medication understanding
		Improving patient satisfaction

## Data Availability

Data was obtained from PubMed (MEDLINE), Ovid Embase, Ovid International Pharmaceutical Abstracts (1970 to April 2025), and Scopus (2012 to 2025).

## Abbreviations

The following abbreviations are used in this manuscript:

BC	Breast cancer
ADR(s)	Adverse drug reaction(s)
DDI	Drug–drug interaction
QOL	Quality of life
BSE	Breast self-examination
RCT	Randomized controlled trial
MTM	Medication therapy management
CMM	Comprehensive medication management
OHT	Oral hormonal therapy
QALYs	Quality-adjusted life years
ER	Emergency room
DRP	Drug-related problem
CSI	Clinically significant interactions
CDTM	Collaborative drug therapy management
CAIT	Cancer and Aging Interdisciplinary Team
CB-PCT	Consultation-Based Palliative Care Team
EPR	Electronic patient record
AET	Adjuvant endocrine therapy
CDK4/6i	Cyclin-dependent kinase 4/6 inhibitor
COVID-19	Corona Virus Disease 2019
HFS	Capecitabine-related hand–foot syndrome
AEs	Adverse events

## Appendix A

### Appendix A.1

**Table A1:** PRISMA-ScR framework Table.

Section	Item	Prisma-ScR Checklist Item	Reported on Page
**TITLE**			
Title	1	Identify the report as a scoping review.	1
**ABSTRACT**			
Structured summary	2	Provide a structured summary that includes (as applicable): background, objectives, eligibility criteria, sources of evidence, charting methods, results, and conclusions that relate to the review questions and objectives.	1
**INTRODUCTION**			
Rationale	3	Describe the rationale for the review in the context of what is already known. Explain why the review questions/objectives lend themselves to a scoping review approach.	1–2
Objectives	4	Provide an explicit statement of the questions and objectives being addressed with reference to their key elements (e.g., population or participants, concepts, and context) or other relevant key elements used to conceptualize the review questions and/or objectives.	2
**METHODS**			
Protocol and registration	5	Indicate whether a review protocol exists; state if and where it can be accessed (e.g., a Web address); and if available, provide registration information, including the registration number.	3
Eligibility criteria	6	Specify characteristics of the sources of evidence used as eligibility criteria (e.g., years considered, language, and publication status), and provide a rationale.	2–3
Information sources	7	Describe all information sources in the search (e.g., databases with dates of coverage and contact with authors to identify additional sources), as well as the date the most recent search was executed.	2–3
Search	8	Present the full electronic search strategy for at least 1 database, including any limits used, such that it could be repeated.	2
Selection of sources of evidence†	9	State the process for selecting sources of evidence (i.e., screening and eligibility) included in the scoping review.	2–3
Data charting process‡	10	Describe the methods of charting data from the included sources of evidence (e.g., calibrated forms or forms that have been tested by the team before their use, and whether data charting was done independently or in duplicate) and any processes for obtaining and confirming data from investigators.	3
Data items	11	List and define all variables for which data were sought and any assumptions and simplifications made.	3
Critical appraisal of individual sources of evidence§	12	If done, provide a rationale for conducting a critical appraisal of included sources of evidence; describe the methods used and how this information was used in any data synthesis (if appropriate).	(-)
Synthesis of results	13	Describe the methods of handling and summarizing the data that were charted.	4
**RESULTS**			
Selection of sources of evidence	14	Give numbers of sources of evidence screened, assessed for eligibility, and included in the review, with reasons for exclusions at each stage, ideally using a flow diagram.	4
Characteristics of sources of evidence	15	For each source of evidence, present characteristics for which data were charted and provide the citations.	4–14
Critical appraisal within sources of evidence	16	If done, present data on critical appraisal of included sources of evidence (see item 12).	(-)
Results of individual sources of evidence	17	For each included source of evidence, present the relevant data that were charted that relate to the review questions and objectives.	4–15
Synthesis of results	18	Summarize and/or present the charting results as they relate to the review questions and objectives.	9–10,14–15
**DISCUSSION**			
Summary of evidence	19	Summarize the main results (including an overview of concepts, themes, and types of evidence available), link to the review questions and objectives, and consider the relevance to key groups.	15–18
Limitations	20	Discuss the limitations of the scoping review process.	18
Conclusions	21	Provide a general interpretation of the results with respect to the review questions and objectives, as well as potential implications and/or next steps.	19
**FUNDING**			
Funding	22	Describe sources of funding for the included sources of evidence, as well as sources of funding for the scoping review. Describe the role of the funders of the scoping review.	19

### Appendix A.2

PubMed(MEDLINE):

(pharmacists[mesh] OR pharmacies[mesh] OR pharmaceutical services[mesh] OR practice patterns, pharmacists’[mesh] OR pharmacist*[tiab] OR pharmacy[tiab] OR pharmacies[tiab] OR “pharmaceutical care”[tiab] OR “pharmaceutical service*”[tiab]) AND (breast neoplasms[mesh] OR “breast cancer”[tiab:~2] OR “breast neoplasm”[tiab:~2] OR “breast neoplasms”[tiab:~2] OR “breast carcinoma”[tiab:~2] OR “mammary cancer”[tiab:~2] OR “breast tumor”[tiab:~2] OR “breast tumors”[tiab:~2] OR “breast tumour”[tiab:~2] OR “breast tumours”[tiab:~2] OR “ductal carcinoma in situ”[tiab] OR “invasive ductal carcinoma”[tiab] OR “lobular carcinoma in situ”[tiab] OR “invasive lobular cancer”[tiab] OR “breast carcinoma in situ”[tiab]) AND English[lang] AND 2012:2025[edat]

Embase

(TITLE-ABS-KEY ((pharmacist* OR pharmacy OR pharmacies OR “pharmaceutical care” OR “pharmaceutical service*”)) AND TITLE-ABS-KEY (breast W/2 (cancer OR neoplasm* OR carcinoma OR tumor* OR tumour*)) OR TITLE-ABS-KEY ((“mammary cancer” OR “ductal carcinoma in situ” OR “invasive ductal carcinoma” OR “lobular carcinoma in situ” OR “invasive lobular cancer” OR “breast carcinoma in situ”)) AND LANGUAGE (english)) AND (LIMIT-TO (PUBYEAR, 2025) OR (LIMIT-TO (PUBYEAR, 2024) OR (LIMIT-TO (PUBYEAR, 2023) OR (LIMIT-TO (PUBYEAR, 2022) OR LIMIT-TO (PUBYEAR, 2021) OR LIMIT-TO (PUBYEAR, 2020) OR LIMIT-TO (PUBYEAR, 2019) OR LIMIT-TO (PUBYEAR, 2018) OR LIMIT-TO (PUBYEAR, 2017) OR LIMIT-TO (PUBYEAR, 2016) OR LIMIT-TO (PUBYEAR, 2015) OR LIMIT-TO (PUBYEAR, 2014) OR LIMIT-TO (PUBYEAR, 2013) OR LIMIT-TO (PUBYEAR, 2012))

Ovid International Pharmaceutical Abstracts

1. (pharmacist* or pharmacy or pharmacies or “pharmaceutical care” or “pharmaceutical service*”).ti,ab.
2. (breast adj2 (cancer or neoplasm* or carcinoma or tumor* or tumour*)).ti,ab.
3. (“mammary cancer” or “ductal carcinoma in situ” or “invasive ductal carcinoma” or “lobular carcinoma in situ” or “invasive lobular cancer” or “breast carcinoma in situ”).ti,ab.
4. 2 or 3
5. 1 and 4
6. limit 5 to (english language and yr = “2012 -Current”)

Scopus

(TITLE-ABS-KEY ((pharmacist* OR pharmacy OR pharmacies OR “pharmaceutical care” OR “pharmaceutical service*”)) AND TITLE-ABS-KEY (breast W/2 (cancer OR neoplasm* OR carcinoma OR tumor* OR tumour*)) OR TITLE-ABS-KEY ((“mammary cancer” OR “ductal carcinoma in situ” OR “invasive ductal carcinoma” OR “lobular carcinoma in situ” OR “invasive lobular cancer” OR “breast carcinoma in situ”)) AND LANGUAGE (english)) AND LIMIT-TO (PUBYEAR, 2025) OR LIMIT-TO (PUBYEAR, 2024) OR LIMIT-TO (PUBYEAR, 2023) OR (LIMIT-TO (PUBYEAR, 2022) OR LIMIT-TO (PUBYEAR, 2021) OR LIMIT-TO (PUBYEAR, 2020) OR LIMIT-TO (PUBYEAR, 2019) OR LIMIT-TO (PUBYEAR, 2018) OR LIMIT-TO (PUBYEAR, 2017) OR LIMIT-TO (PUBYEAR, 2016) OR LIMIT-TO (PUBYEAR, 2015) OR LIMIT-TO (PUBYEAR, 2014) OR LIMIT-TO (PUBYEAR, 2013) OR LIMIT-TO (PUBYEAR, 2012))
